# Airway Microbiota in Patients With Synchronous Multiple Primary Lung Cancer: The Bacterial Topography of the Respiratory Tract

**DOI:** 10.3389/fonc.2022.811279

**Published:** 2022-04-12

**Authors:** Kai Qian, Yi Deng, William S. Krimsky, Yong-Geng Feng, Jun Peng, Yong-Hang Tai, Hao Peng, Li-Hong Jiang

**Affiliations:** ^1^ Department of Thoracic Surgery, The First People’s Hospital of Yunnan Province, Kunming, China; ^2^ The Affiliated Hospital of Kunming University of Science and Technology, Kunming, China; ^3^ Faculty of Life and Biotechnology, Kunming University of Science and Technology, Kunming, China; ^4^ Department of Pulmonary and Critical Care Medicine, The First People’s Hospital of Yunnan Province, Kunming, China; ^5^ Chief Medical Officer, Gala Therapeutics, San Carlos, CA, United States; ^6^ Department of Thoracic Surgery, Institute of Surgery Research, Daping Hospital, Army Medical University, Chongqing, China; ^7^ School of Physics and Electronic Information, Yunnan Normal University, Kunming, China

**Keywords:** electromagnetic navigation bronchoscopy, microbiota (16S), synchronous multiple primary lung cancer, bronchoalveolar lavage, bacterial topography

## Abstract

Microbes and microbiota dysbiosis are correlated with the development of lung cancer; however, the airway taxa characteristics and bacterial topography in synchronous multiple primary lung cancer (sMPLC) are not fully understood. The present study aimed to investigate the microbiota taxa distribution and characteristics in the airways of patients with sMPLC and clarify specimen acquisition modalities in these patients. Using the precise positioning of electromagnetic navigation bronchoscopy (ENB), we analyzed the characteristics of the respiratory microbiome, which were collected from different sites and using different sampling methods. Microbiome predictor variables were bacterial DNA burden and bacterial community composition based on 16sRNA. Eight non-smoking patients with sMPLC in the same pulmonary lobe were included in this study. Compared with other sampling methods, bacterial burden and diversity were higher in surface areas sampled by bronchoalveolar lavage (BAL). Bacterial topography data revealed that the segment with sMPLC lesions provided evidence of specific colonizing bacteria in segments with lesions. After taxonomic annotation, we identified 4863 phylotypes belonging to 185 genera and 10 different phyla. The four most abundant specific bacterial community members detected in the airway containing sMPLC lesions were *Clostridium, Actinobacteria, Fusobacterium*, and *Rothia*, which all peaked at the segments with sMPLC lesions. This study begins to define the bacterial topography of the respiratory tract in patients with sMPLC and provides an approach to specimen acquisition for sMPLC, namely BAL fluid obtained from segments where lesions are located.

## Introduction

With improved imaging techniques, an increasing number of patients with synchronous multiple lung nodules are being diagnosed (1). Among these patients, coexisting primary lung cancers are called synchronous multiple primary lung cancer (sMPLC), characterized by at least two primary tumors simultaneously identified in the ipsilateral or contralateral lung. At present, surgical resection is recommended for patients with certain sMPLC, but the prerequisite for this technique is a clear diagnosis of sMPLC before surgery. Histological differences between multiple nodules are reliable indicators of sMPLC; however, it is challenging to differentiate a second primary cancer from a satellite, metastatic, or recurrent lesion of the original tumor if multiple tumors are histologically the same or similar. To better define the relationship among multiple lesions in the lung, alternative approaches using novel molecular testing, such as immunohistochemical and molecular analyses, have been proposed in recent studies (2). Moreover, the clonality of multiple lesions can be diagnosed based on array comparative genomic hybridization analysis, loss of heterozygosity (LOH) analysis, or the occurrence of somatic mutations in tumor suppressor genes or oncogenes. Contrary to this, some studies have profiled the gene mutations of sMPLC and found that many cases of sMPLC did not harbor the driver mutations that commonly occur in lung adenocarcinoma (3).

Previous studies have suggested that dysbiosis of the microbiota may influence the pathogenesis, progression, and outcome of lung cancer at multiple levels by affecting metabolic, inflammatory, or immune pathways (4–6). Improving knowledge about the interplay between the lung microbiome and lung cancer can promote the development of innovative strategies for the personalized treatment of sMPLC, and potentially for its prevention as well. In this process, specifically assessing the core microbiota in sMPLC and integrating this with information regarding the microbiota surrounding the tumors is a novel method for evaluating this unexplored area. Nevertheless, many factors can influence the investigation of pulmonary microbiota. For example, the microbiota composition differs according to the sampling site (7), sampling period, and sampling applications. Additionally, the ecosystem of lung microbiota has all the features of a complex adaptive system: diverse entities interacting with each other within a common space, showing interdependent actions, and possessing the capacity to adapt to changing conditions (8). As far as we know, only one study has focused on patients with synchronous multiple ground-glass nodules (SM-GGNs) who have two GGN lesions (9); however, the respiratory microbiome sampling method used for sMPLC is unknown. Therefore, distinct analytical approaches to further reveal the interplay of these complex adaptive systems in sMPLC are needed.

In this study, we used microbial diversity analysis to systematically characterize the bacterial microbiota in different sites of the airway in patients with sMPLC and assess and compare different methods of specimen acquisition. Herein, we propose a systematic method to analyze bacterial communities of the respiratory tract in sMPLC, thereby further expanding the study of respiratory microbiologic diversity utilizing electromagnetic navigational bronchoscopy.

## Materials and Methods

### Patients

Currently, there are no clear guidelines available for the diagnosis of sMPLC. We used the Antakli modifications as the basic criteria for the definition of sMPLC (10). The details are as follows: (1) one lesion in each patient must be diagnosed as an adenocarcinoma; (2) the lesions must be in anatomically distinct regions; (3) there must be no systemic metastases; (4) there must be no mediastinal spread as confirmed by postoperative pathology; and (5) patients must possess two or more of the following: (i) one tumor diagnosed as an adenomatous tumor, i.e., minimally invasive carcinoma, or invasive AD (3), and another tumor diagnosed as either squamous carcinoma or small cell lung cancer; (ii) different driver gene mutations; and (iii) different biomarker patterns (11). The inclusion criteria were as follows: (1) patients diagnosed with sMPLC, as previously described, and all lesions located in the right upper lobe (RUL); (2) aged ≥18 years; (3) no contraindications for pulmonary surgery, i.e., distant metastasis, bleeding tendency, blood clotting disorders, cardiopulmonary insufficiency, severe arrhythmia or hypertension, pulmonary hypertension, and acute respiratory infection; (4) no other prior therapies; (5) expected survival >2 months; (6) without a history of other primary malignant tumors; (7) agreeing to participate in this study. Exclusion criteria included patients with poorly controlled diabetes, prior cerebrovascular events, active second malignancy, uncontrolled concomitant illness, or other conditions that might affect their participation in the study. The study protocol was reviewed and approved by the Research Ethics Board of Daping Hospital [reference no. 20200009]. This study was carried out in accordance with the Declaration of Helsinki, and written informed consent was obtained from all participants.

### Sample Acquisition and Processing

**Figure 1:**
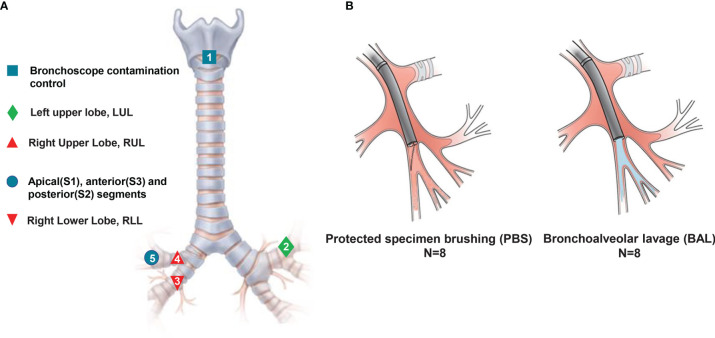
Experimental design model. Eight patients with no infectious respiratory diseases underwent serial sampling by ENB, and a series of approaches such as spatiotemporal interleaving was used to prevent cross-contamination. **(A)** Sampling methods and locations. Numbers refer to the sampling order. **(B)** Schematic diagram of methods: avoiding contact with airway mucosa when brushing a discrete area of the airway mucosa in PSB (left), and sampling airways distal to the wedged bronchoscope in BAL (right).

All of the patients received general anesthesia. Oral cavity microbiota was sampled before intubation, and then the bronchoscope was quickly advanced to the vocal cords without suctioning. The sequence of sampling is detailed in **Figure 1**. After exploration with a bronchoscope, a locatable guide was inserted *via* an extended working channel (2.8 × 1050 mm), and the virtual and actual bronchoscopy images were matched and registered using a Super-D electromagnetic navigation system (AAS000161-02; USA) (12). Prior to any microbiota sampling procedure, a control saline sample was collected *via* aspiration through the bronchoscope. For bronchoalveolar lavage (BAL), this was performed with the instillation of 200 mL of sterile isotonic saline (13). A cell count of the BAL specimen was performed using the pooled BAL fluid *via* hemocytometry at the Kunming University of Science and Technology Clinical Laboratory, and then the BAL was frozen and stored at −80°C until processing.

In order to prevent the contamination caused by the process of sampling, we used three prevention methods. First, we used rational sampling to avoid contamination. Due to the different anatomical structure between left and right trachea, we explored the left upper lobe trachea (without lesions) before the right side (with lesions). In different lobes on the same side of the lung, we prioritized exploring the lower lobe bronchi, which are prone to contamination due to gravity. Second, we avoided contamination by maintaining the sterility of equipment. We replaced the contaminated bronchoscope during the contemporaneous sampling in different locations. Third, we used an interval sampling time to avoid contamination. Mutual contamination is unavoidable when obtaining specimens from lobe bronchus and segments in the same lobe. Therefore, we used a one-week interval to avoid cross-contamination from same lobe and segment.

### 16S DNA Sequencing and Sequence Data Analysis

DNA was extracted from saliva samples using a QIAamp DNA Microbiome Kit (Qiagen, Hilden, Germany), per the manufacturer’s recommendations. This kit can be used to effectively deplete host DNA and fully extract DNA from bacteria (14). V3 and V4 hypervariable regions of bacteria and archaea 16sRNA were amplified using forward primers containing the sequence “CCTACGGRRBGCASCAGKVRVGAAT” and reverse primers containing the sequence “GGACTACNVGGGTWTCTAATCC.” At the same time, indexed adapters were added to the ends of the 16S rDNA amplicons (15). We carried out 2×300 bp double-end sequencing, according to the Illumina MiSeq (Illumina, San Diego, CA, USA) instruction manual, and the sequence information was read using MiSeq Control Software. Quality filtering on joined sequences was performed, and sequences that did not fulfill the following criteria were discarded: sequence length <200 bp, no ambiguous bases, and mean quality score ≥20. Then, the sequences were compared with the reference database (RDP Gold database) using the UCHIME algorithm to detect the sequences, and chimeric sequences were removed (16).

### Quality Control

All samples were processed by the same researcher (Yi Deng) under the same experimental conditions, and staff were blinded to the sample status. During electromagnetic navigation bronchoscopy (ENB), independent aseptic extended working channels were used to reach each sampling site. Separation and extraction of all samples, saliva and BAL, were carried out in an aseptic laminar flow hood, and all steps were taken to ensure an aseptic operation. Negative control samples (without a DNA template) were used to detect possible reagent and environmental contamination in all sequencing batches. Furthermore, all samples were sequenced in the same batch.

### Diversity Analysis Statistics

We applied alpha and beta diversity statistics in the QIIME2 package to compare communities at both microbiota and microbiome levels. For alpha diversity, we counted the number of distinguishable taxa (OUTs) in each sample as species richness. The independent samples *t*-test, correction Student *t*-test, and Wilcoxon rank-sum test were used for the Chao1, Shannon, and Simpson indices, respectively. Heat maps were generated in R with the ComplexHeatmap package (17). For beta diversity, we determined the significance of differences in community composition using permutational multivariate analysis of variance (PERMANOVA) and analysis of similarities (ANOSIM) with 1,000 permutations and constructed both linear and quadratic mixed models using the lmer function in R packages lme4 and lmerTest. Density plots were generated on the basis of the Bray–Curtis similarity measure using the density function in the R Stats package (18). Principal coordinate analysis (PCoA) was used to estimate the similarity between samples. LEfSe analysis was used to identify differences between two groups of bacteria at all levels. Metastats difference analysis was used to identify differences in species abundance difference at genus level between groups.

## Results

### Study Population

A total of eight patients with sMPLC were included in this study; no patient was immunosuppressed or had an active respiratory infection. All patients received video-assisted thoracoscopic surgery (VATS) therapy. Detailed clinical features are summarized in **Table 1**. The mean patient age was 53.9 ± 9.1 years, with five (62.5%) women and three (37.5%) men. All tumors were located in the RUL, and all patients were non-smokers. There was a total of 17 lesions, including five lesions in apical segment (S1), seven lesions in posterior segment (S2), and five lesions in anterior segment (S3). The clinical characteristics of these patients are given in **Table 1**. In total, 19,392 ± 861 (mean ± standard deviation) sequence reads per specimen were obtained, and we did not exclude any specimen because of insufficient sequences.

**Table 1:** Clinical characteristics of patients.

Patient	Race	Gender	Age	Smoking history	Tumor number	Tumor location	Surgical methods
P1	Chinese	Female	56	None	2	S1+S2	lobectomy
P2	Chinese	Male	63	None	3	S2+S3	lobectomy
P3	Chinese	Female	71	None	2	S1+S2	lobectomy
P4	Chinese	Female	58	None	2	S1+S3	lobectomy
P5	Chinese	Male	63	None	2	S1+S3	lobectomy
P6	Chinese	Female	52	None	2	S2+S3	lobectomy
P7	Chinese	Male	49	None	2	S2	lobectomy
P8	Chinese	Female	55	None	2	S1+S2	lobectomy

AAH, atypical adenomatous hyperplasia; AIS, adenocarcinoma in situ; MIA, minimally invasive adenocarcinoma; AD, invasive adenocarcinoma.

### Selection of Sample Acquisition Modalities in sMPLC

**Figure 2:**
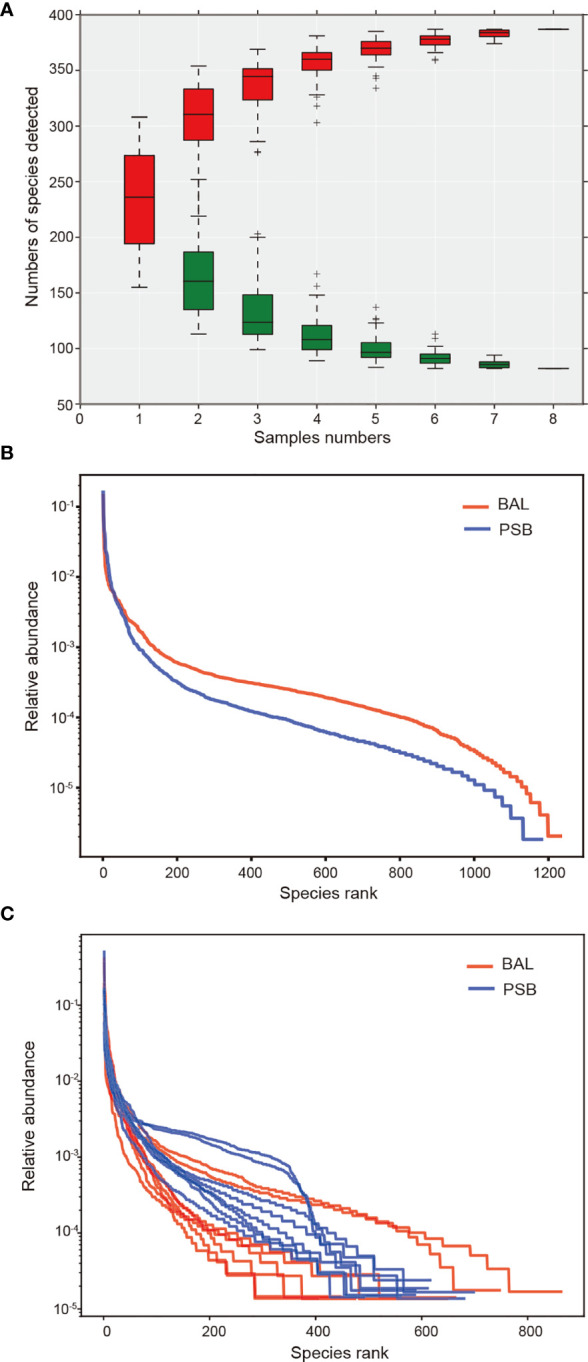
Changes in microbiome diversity in microbial acquisition modalities. In this figure, we measured the relationship between the number of samples and the number of species annotated using a species relative abundance accumulation curve. **(A)** The relative abundance accumulation curve of species shows that the eight samples were sufficient for further research. The abscissa represents the grouping; the vertical axis represents the distance; boxes of different colors represent each grouping, with red representing PSB and green representing BAL. Rank abundance curves and diversity indexes between BAL (n = 8) and PSB (n = 8) groups **(B, C)**, confirming significant differences in species diversity between groups. Species abundance is reflected by the length of the curve on the horizontal axis, and the uniformity of species composition is reflected by the shape of the curve.

We conducted an alpha diversity analysis in an unaffected location, the left upper lobe, to further understand species richness and microbiome structure between two sampling modalities, i.e., protected specimen brushing (PSB) and BAL. We measured the relationship between the number of samples and the number of species annotated using a species relative abundance accumulation curve. We found that the curve tended to be flat in both groups (**Figure 2A**), indicating that the sample was sufficient for further analysis (19). The alpha diversity indexes were significantly greater in BAL specimens than in distal PSB specimens from the extended working channel during ENB (**Figures 2B, C**; **Table 2**). We speculated that these results reflect differences in the surface areas sampled. PSB samples were taken from the extended working channel during ENB, with approximately 1 cm^2^ of the airway mucosa; BAL fluid was from a wedged subsegment sample approximately 1/40 of the total surface area of the lungs (20) or approximately 17,500 cm^2^ (21). Hence, the larger surface area sampled using BAL permits the detection of a greater bacterial signal, with minimal influence from bronchoscopic contamination. The results regarding bacterial burdens detected *via* PSB and BAL were comparable to the values reported in previous studies (22, 23).

**Table 2:** Alpha diversity analysis and statistical analysis.

Methods	PSB	BAL	P-value
Chao1	179.07	189.44	0.219^a^
Ace	180.08	187.58	0.346^a^
Shannon	4.55	4.66	0.652^b^
Simpson	0.88	0.92	0.624^c^

The first two columns are the mean values of the two groups in these four indices. a. Independent sample t-test; b. Correction Student’s t-test; c. Wilcoxon rank-sum test.

### Differences in Microbial Community Composition and in Measures of Diversity Between Various Airway Locations

**Figure 3:**
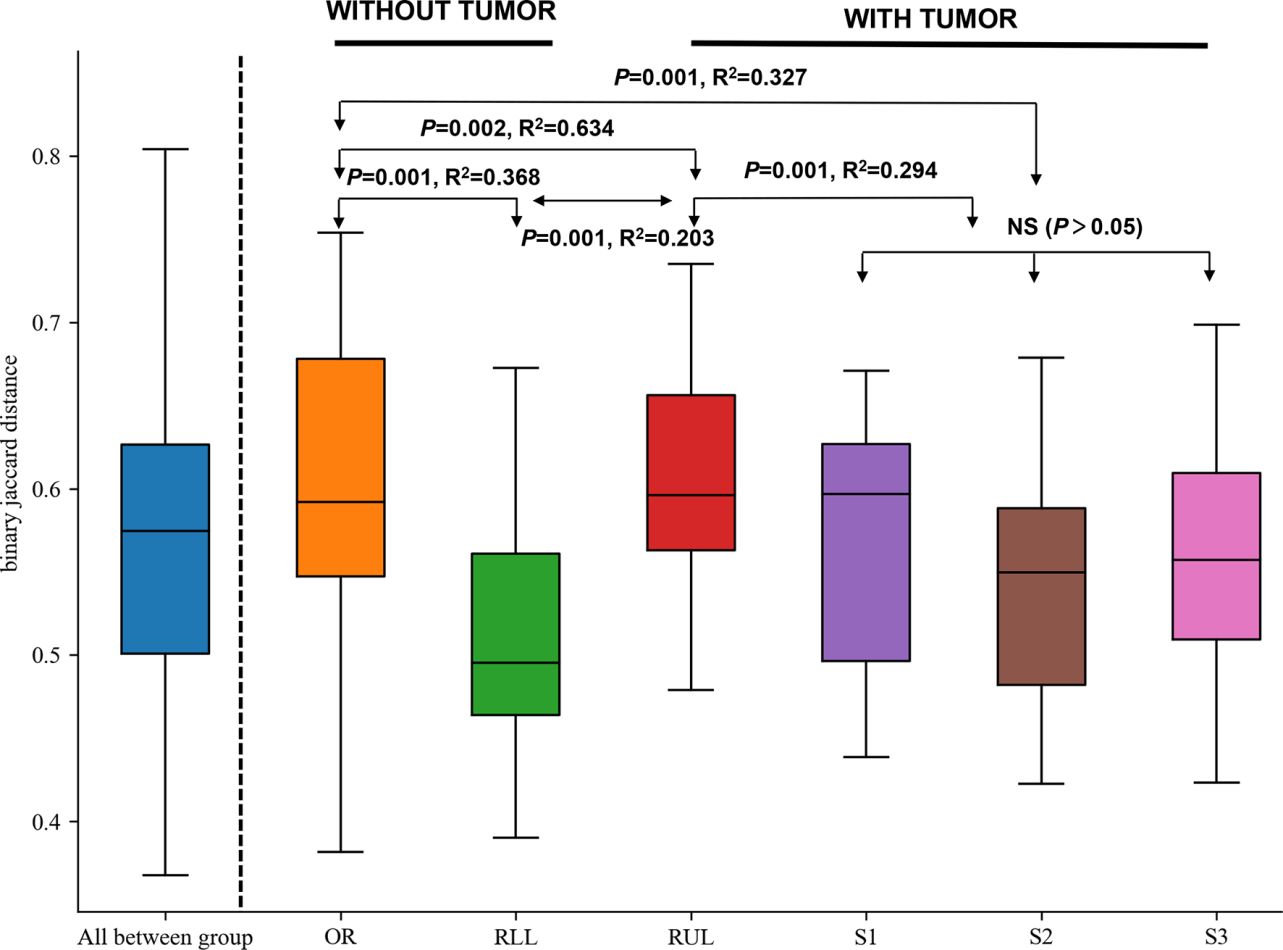
Formal comparisons between regions evaluated by analysis of similarities (ANOSIM) revealed significant differences between the OR and consecutive sampling regions in the respiratory tract. NS, not significant; OR, oral cavity; RUL, right upper lobe; RLL, right lower lobe; S1, apical segment; S3, anterior segment; and S2, posterior segment.

On the basis of the abovementioned investigation of sampling modalities, we used PERMANOVA to systematically compare indices of bacterial colonization at the oral cavity (OR); right upper lobe (RUL) sites; apical (S1), posterior (S2), and anterior (S3) segments; and right lower lobe (RLL) sites (**Figure 1**). Comparisons between the bacterial assemblages at OUT level across the six different regions of the airway tract revealed that the bacterial structures were significantly different between tumor regions (RUL) and non-tumor regions (RLL) (PERMANOVA R^2 ^= 0.203, P=0.001; ANOSIM global R=0.696, P=0.001) (**Figure 3** and **Table S1**). Both PERMANOVA and ANOSIM revealed significant differences between the OR and consecutive sampling regions in the respiratory tract, i.e., RUL, RLL, and lung segments of the RUL (**Figure 3** and **Table 3**); all of these were confirmed using PCoA (**Figure S1**). Additionally, no differences were observed between bacterial communities in the samples retrieved from different segments within the RUL, even at phylotype level (**Figure 3**).

**Table 3:** PERMANOVA and ANOSIM analysis for community structures.

Group	PERMANOVA		ANOSIM	
	R^2^	P-value	R	P-value
OR and RUL	0.634	0.002	0.416	0.001
OR and RLL	0.368	0.001	0.721	0.001
RUL and S1	0.294	0.001	0.329	0.001
RUL and S2	0.374	0.001	0.888	0.001
RUL and S3	0.197	0.002	0.313	0.001
RUL and RLL	0.244	0.001	0.829	0.003

### Relative Contributions of Mucosal Dispersion to Microbial Immigration in sMPLC

**Figure 4:**
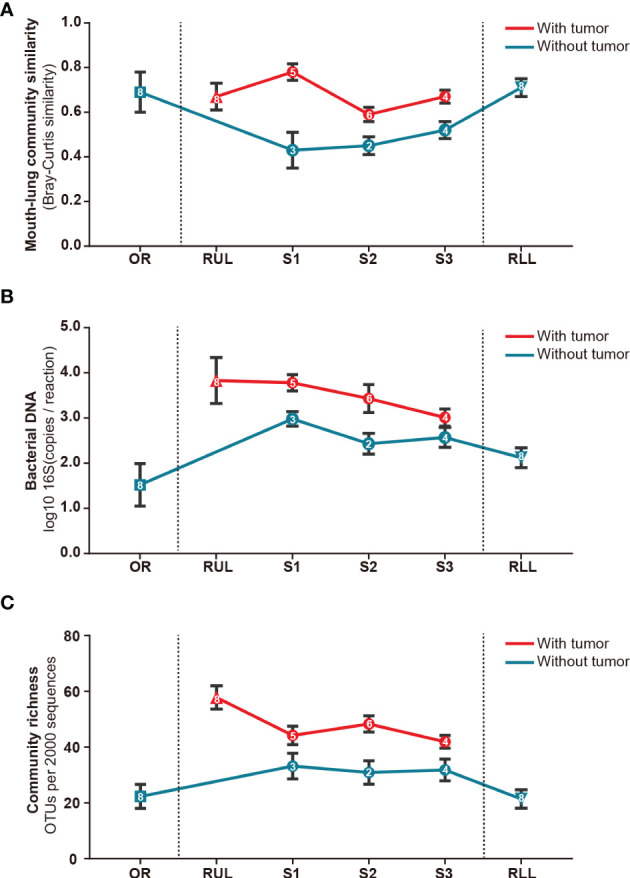
Bacterial topography in sMPLC. To clarify whether there was mouth–lung bacterial immigration to the RUL or specific colonizing bacteria in the RUL, we quantified specimens from different sites using mouth–lung community similarity (Bray–Curtis similarity). **(A)** Bacterial DNA (log10 number of 16S copies per reaction determined by real-time qPCR); **(B)** community richness (number of OTUs per 2,000 sequences); **(C)** connecting lines (red: with tumor, blue: without tumor) of bacterial expression levels in specimens from different sites (square: oral cavity, triangle: lung lobe, circle: pulmonary segments) of the respiratory tract exhibiting less evidence of mouth–lung immigration than RUL specimens (*P <* 0.001, paired Student’s *t-*test). Indices of mouth–lung immigration in airway PSB samples were nonlinear. Patients with sMPLC showed regional differences in flora caused by non-aspiration. Data are mean ± SEM (n *=* 8). RUL, right upper lobe; RLL, right lower lobe; SEM, standard error of mean.

Next, we analyzed the bacterial topographic data to determine the relative contributions of mucosal dispersion to microbial immigration in sMPLC. In this study, all included patients had multiple primary tumor lesions in the RUL. To determine whether the difference in taxa arises from mouth–lung immigration or specific colonizing bacteria in the RUL, we used mouth–lung immigration (mouth–lung community similarity, total bacterial burden, and community richness) to assess whether pulmonary bacterial immigration exists in the RUL (8, 24). In this process, when collective community structures were examined using PERMANOVA and ANOSIM (25), we confirmed that oral specimen communities were distinguishable from communities in RUL specimens (P<0.05) (**Figure 4**). As predicted, specific taxa colonized specific bronchial mucosal surfaces. Otherwise, indices of the different taxa in BAL samples from the oral cavity mucosal dispersion colonized along the airways. This ecological trend could be seen most distinctly in mouth–lung similarity (Bray–Curtis distance) (**Figure 4A**), which failed to fit the linear relationship predicted for dispersion along the bronchial mucosa (P>0.05). However, some patients exhibited evidence of bacterial immigration at the lobe and segment sites with tumors (see bimodal density plots for these sites in **Figures 4B, C**). Taken together, the bacterial topography data from BAL samples were inconsistent with contiguous bronchial mucosa being the primary immigration route, despite the dissimilar communities present at the different segments of the RUL.

### Taxa Characteristics of Tumor and Non-Tumor Regions

**Figure 5:**
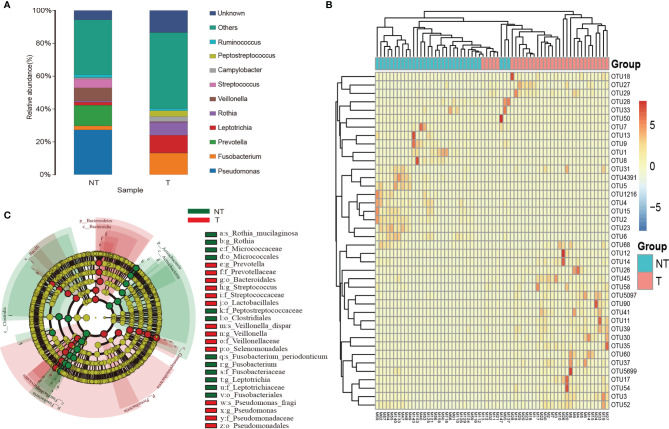
Taxa characteristics of tumor and non-tumor regions. **(A)** Species distribution histogram. Color represents species, and the length of the color block represents relative abundance ratio of the species. **(B)** Heat maps of the two groups of bacteria. **(C)** The circle radiating from inside to outside represents the classification from phylum to genus level. Each small circle represents a classification at that level in different classification levels. The diameter of the small circle is proportional to the relative abundance. c_ is class, o_ is order, f_ is family, and g_ is genus; T, pulmonary segments with tumor; NT, pulmonary segments without tumor.

Sequences detected in procedural specimens were significantly distinct from sequences detected in specimens from tumor sites and sites without tumor (P<0.05 for all comparisons) (**Figure S1**). The three most abundant taxa detected in sequences from tumorous locations were classified as *Fusobacterium* sp. (operational taxonomic unit [OTU]00012), *Leptotrichia* sp. (OTU00054), and *Rothia* sp. (OTU00035), comprising 31.8 ± 6.2% (mean ± standard error of the mean [SEM]) of all sequences from tumorous locations (**Figure 5A** and **Table S2**). In contrast, these three OTUs collectively made up 11.3 ± 1.6% of sequences from RUL specimens. The three most abundant taxa detected in sequences from non-tumorous locations were classified as *Pseudomonas* sp. (OTU00013), *Prevotella* sp. (OTU0007), and *Veillonella* sp. (OTU00031), comprising 67.3% of RUL sequences (**Figures 5A, B** and **Table S2**), consistent with previous studies of normal lobes (24, 26). According to the Metastats difference analysis and LEfSe analysis (the default was analyzed at genus level), the four most abundant specific bacterial community members detected in the airway containing tumor were *Clostridium, Actinobacteria, Fusobacterium*, and *Rothia*, (**Figure 5C** and **Table S3**), and the differences between the four bacteria in the two groups were significant (P<0.05). Moreover, each peaked in segments with tumors and the RUL bronchus, with significantly smaller fractions in both normal segments of the RUL and RLL airway (P<0.01) (**Figure S2**). The topographic relative abundance of these bacterial community members was also consistent with the location of sMPLC (**Figure 4**).

## Discussion

Investigative bronchoscopy is increasingly being incorporated into multicenter observational trials to assess potential links between the microbiome to intermediate biomarkers of disease activity and progression (27–29). Similar to various pulmonary diseases, lung bacterial communities show pronounced anatomic heterogeneity in sMPLC (4); this consideration takes on special importance when attempting to define how interactions between hosts and microbes might contribute to pathogenesis in different airway locations.

Additionally, vetted strategies for obtaining specimens remains another problem relative to the study of lung microbiology, especially given the necessity of avoiding inaccurate positioning and contamination with other microorganisms. In principle, contamination comes from two major sources: passage of the bronchoscope through the airway (30) and bacterial DNA present in laboratory reagents (31). Since the pathogenesis of sMPLC is currently unclear, the necessity of clarifying the biological microenvironment around these lesions is of substantive importance.

We previously demonstrated that the use of the ENB technique is a safe, effective method to increase the diagnostic accuracy of peripheral lung lesions (32). Verifying that sampling by ENB can be used reliably to study the microbiome of patients with sMPLC has several potentially important implications. In this study, the successful application of ENB to locate the extended working channel to reach the lung segment that contained the lesion appears to confirm that there is no difference in diversity between PSB and BAL obtained from the segment by ENB, which further lays a foundation for the study of sMPLC microbial diversity.

Comparison of the detected communities showed that BAL specimens exhibited a greater abundance of RUL immigration than did every segment of the RUL (S1, S2, and S3) (P<0.05 for all three measurements) (**Figure 3**). We interpret these results as reflecting differences in the surface areas sampled, just as we confirmed that BAL specimens exhibited a greater abundance of mouth–lung immigration than did distal PSB specimens (24). Hence, even though a difference in mucosal density is expressed in the different segments of the same lobe, the larger surface area sampled *via* BAL permits the detection of greater bacterial abundance, with minimal influence from bronchoscopic contamination. We found that there was no significant difference in the flora of the non-tumor segment in the lobe with sMPLC. Based on our results, when studying the microbial diversity in sMPLC, we believe that the adjacent lung segments in the same lobe can be used as a negative control.

A further notable finding of our study was the significant difference of community richness and genus type heterogeneity between tumor sites and non-tumor sites in sMPLC. At the genus level, *Fusobacterium*, *Leptotrichia*, and *Rothia* preferentially colonized the mucosa of airways with tumors, whereas *Pseudomonas*, *Prevotella*, and *Veillonella* were more abundant in segments without tumor than in those with tumor sites in the RUL. The observation that bacterial communities in segments are distinct re-emphasizes the need for selective sampling, given the distinction between the bacterial communities described.

Our study provides evidence of site-specific enrichment by reproducing bacteria in sMPLC. The systematic analysis performed at each taxonomic rank forms the basis for detailed characterization of the microbial function in a specific anatomical region (33). We identified sMPLC-specific taxa that were distinct from segment taxa detected in normal lobes or segments without tumor. Our experimental design, with meticulous collection of control specimens and prevention of contamination from other locations that is consistent with numerous previous studies, showed that the microbial signal detected in the airway and alveolar specimens is not an artifact of oral contamination. As additional evidence, we found *Fusobacterium* in segments with tumor, which is inconsistent with the primary source of microbial immigration, i.e., *Prevotella* species (23, 34). However, lobes or segments with sMPLC lesions through which a bronchoscope may pass when using non-protected BAL may contaminate samples; therefore, carrying out protective sampling is necessary. We strongly recommend the use of protective endotracheal microbial sampling methods in patients with sMPLC, such as ENB, to reach the exact lung segment where the lesion is located or the use of a slim extended working channel so as to prevent contamination.

Here, we provide a comprehensive, high-resolution description of the biodiversity and distribution of microbial communities along the airway of patients with sMPLC. Previously, a systematic review focusing on characteristics and prognosis after surgical treatment of sMPLC revealed that age, sex, smoking status, tumor size, and surgical methods are important prognostic factors for patients with sMPLC after surgery (35). The abovementioned clinical factors were all taken into account in this study. Nevertheless, several points need further clarification. First, considering the importance of clarifying the characteristics of different parts of sMPLC, we strictly limited the patients included in this study to those with lesions in the RUL. Thus, subject enrollment was limited to eight patients, but we do anticipate to increasing this number substantively in future studies. Second, although we demonstrated the existence of different taxa within the sMPLC, understanding the underlying mechanisms that mediate these complex taxa differences remains a crucial factor for favorably modulating this difference. Third, in this study, we used ENB technology and a series of approaches such as spatiotemporal interleaving to prevent cross-contamination. This significantly minimized, but did not eliminate, the risk of cross-contamination.

In conclusion, ENB was used to assess and characterize flora in different parts of the respiratory tract of patients with sMPLC. Furthermore, different methods of specimen collection were also evaluated for obtaining respiratory tract flora samples from these patients. We believe that these results provide a sound strategy for conducting future studies on the microbial characteristics of patients with sMPLC.

## Data Availability Statement

The raw data presented in this study has been deposited in the GEO repository, accession number GSE200111. Further inquiries can be directed to the corresponding authors.

## Ethics Statement

The studies involving human participants were reviewed and approved by Research Ethics Board of Daping Hospital [reference no. 20200009]. The patients/participants provided their written informed consent to participate in this study.

## Author Contributions

Conception and design: KQ, WK, and YD. Administrative support: L-HJ and HP. Provision of study materials or patients: KQ, YD, and JP. Collection and assembly of data: Y-GF, KQ, and JP. Data analysis and interpretation: KQ and YD. Manuscript writing: All authors. All authors contributed to the article and approved the submitted version.

## Funding

This study is funded by the Applied Basic Research Foundation of Yunnan Province, 202001AY070001-299, and the First People’s Hospital of Yunnan Province, 2021LCZXXF-HX06.

## Conflict of Interest

The authors declare that the research was conducted in the absence of any commercial or financial relationships that could be construed as a potential conflict of interest.

## Publisher’s Note

All claims expressed in this article are solely those of the authors and do not necessarily represent those of their affiliated organizations, or those of the publisher, the editors and the reviewers. Any product that may be evaluated in this article, or claim that may be made by its manufacturer, is not guaranteed or endorsed by the publisher.

## Supplementary Material

Supplementary Figure 1
**(A, B)** Differences in bacterial community structures along the airways (RUL, right upper lobe; RLL, right lower lobe) of eight MPLC individuals, assessed by principal coordinates analysis (PCoA) based on the Bray–Curtis similarity matrix. **(C)** Non-Metric Multi-Dimensional Scaling (NMDS) analysis of the compositional profiles stratify. A stress value less than 0.2 indicates that the model grouping is reliable.

Supplementary Figure 2 The abundance distribution of the four strains with the largest difference between the tumor and non-tumor groups is shown. The abscissa represents the classification strains’ name in different sites, and the ordinate is the relative abundance of the strain.

## Abbreviations

ANOSIM, analysis of similarities; BAL, bronchoalveolar lavage; ENB, electromagnetic navigation bronchoscopy; OR, oral cavity; PSB, protected specimen brushing; PERMANOVA, permutational multivariate analysis of variance; RUL, right upper lobe; RLL, right lower lobe; sMPLC, synchronous multiple primary lung cancer; SM-GGNs, synchronous multiple ground-glass nodules; VATS, video-assisted thoracoscopic surgery.
