# Supplementary figures and images for: Airway Microbiota in Patients With Synchronous Multiple Primary Lung Cancer: The Bacterial Topography of the Respiratory Tract
Source: Front Oncol. 2022 Apr 12;12:811279. doi: 10.3389/fonc.2022.811279 (PMC9041701; doi:10.3389/fonc.2022.811279)

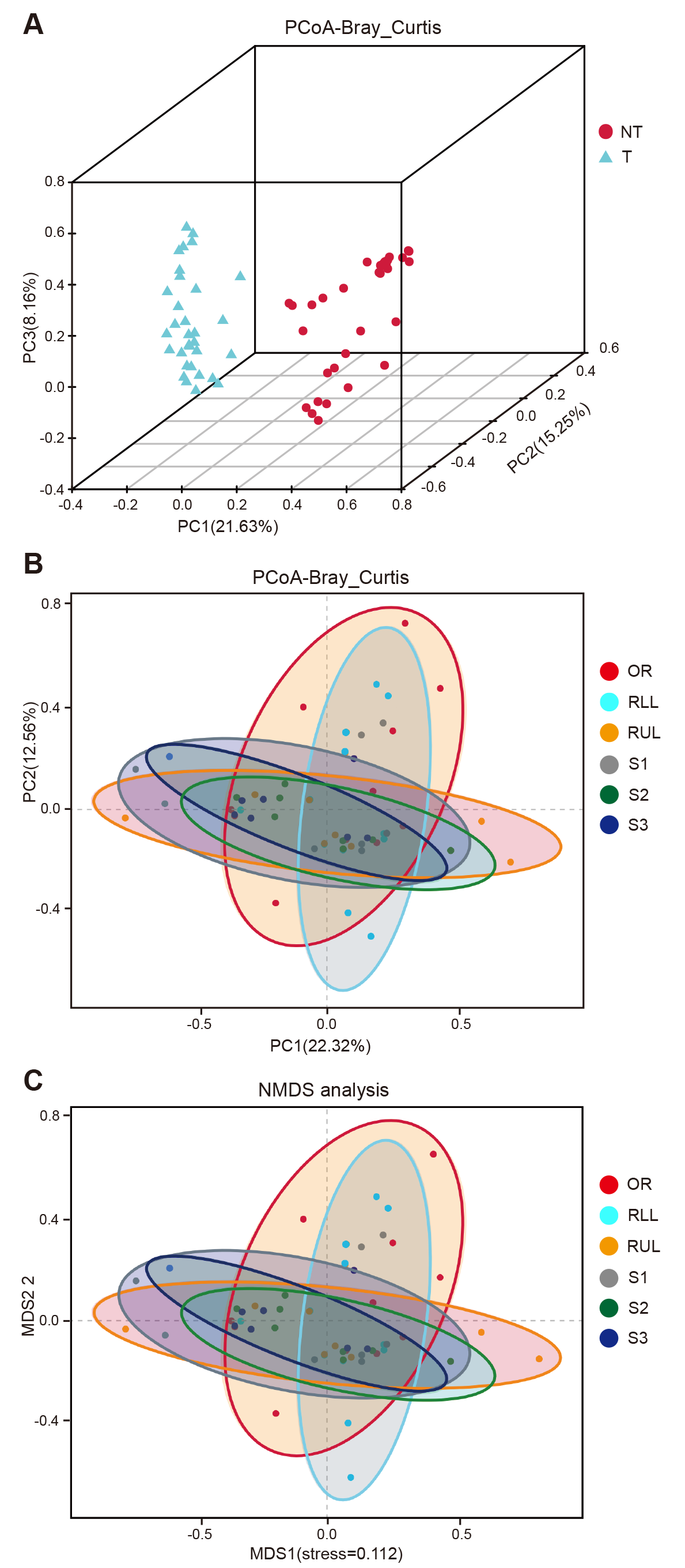

Supplement: Supplementary Figure 1 — (A, B) Differences in bacterial community structures along the airways (RUL, right upper lobe; RLL, right lower lobe) of eight MPLC individuals, assessed by principal coordinates analysis (PCoA) based on the Bray–Curtis similarity matrix. (C) Non-Metric Multi-Dimensional Scaling (NMDS) analysis of the compositional profiles stratify. A stress value less than 0.2 indicates that the model grouping is reliable. [file Image_1.tif]

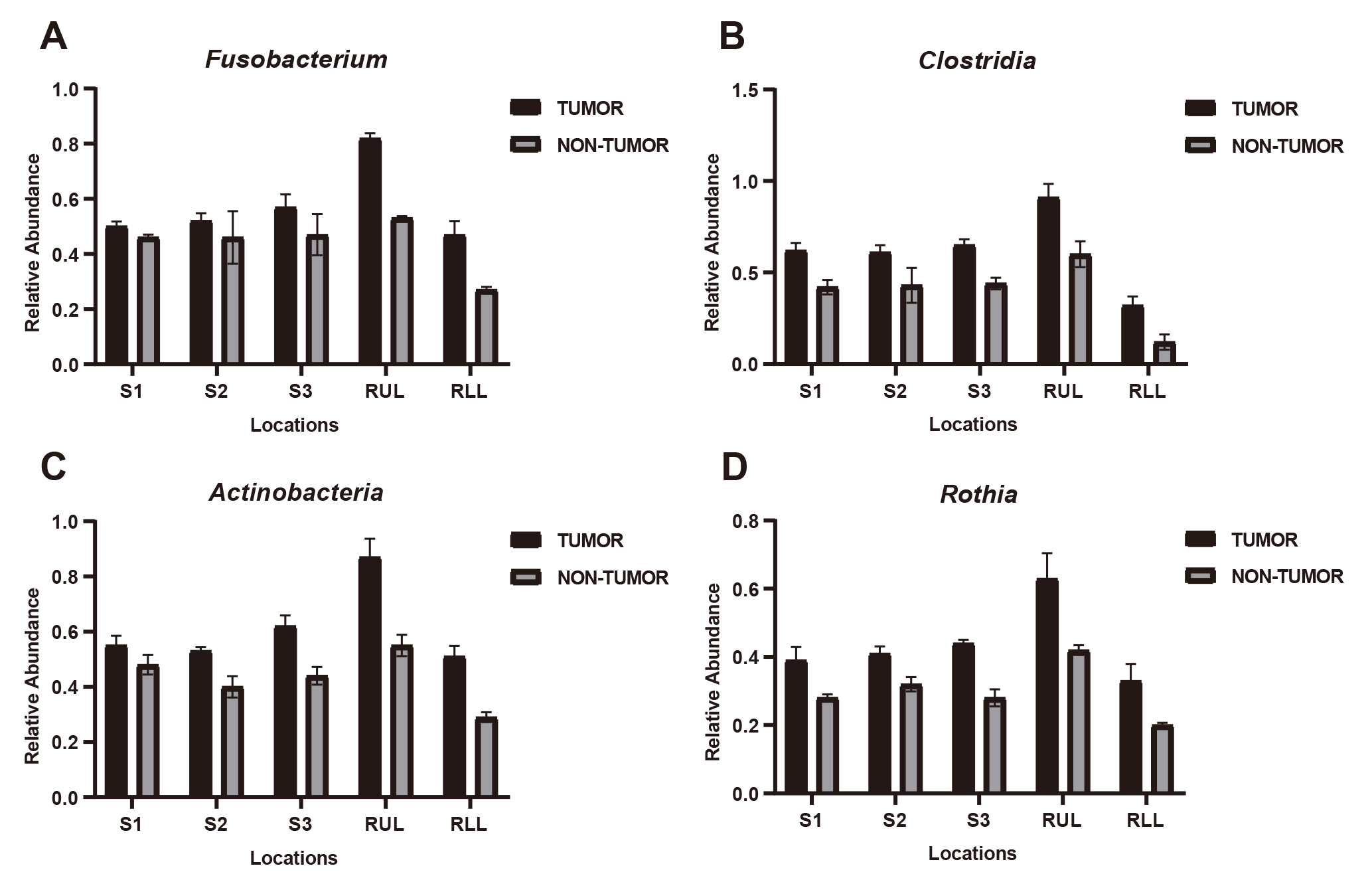

Supplement: Supplementary Figure 2 — The abundance distribution of the four strains with the largest difference between the tumor and non-tumor groups is shown. The abscissa represents the classification strains’ name in different sites, and the ordinate is the relative abundance of the strain. [file Image_2.tif]
